# *N*′-(5-Bromo-2-hydr­oxy-3-methoxy­benzyl­idene)-4-hydr­oxy-3-methoxy­benzohydrazide dihydrate

**DOI:** 10.1107/S1600536809033522

**Published:** 2009-08-29

**Authors:** Jiu-Fu Lu, Suo-Tian Min, Hong-Guang Ge, Xiao-Hui Ji, Yue-Fei Bai

**Affiliations:** a School of Chemistry and Environmental Science, Shaanxi University of Technology, Hanzhong 723000, People’s Republic of China; b The School of Pharmaceutical Engineering, Shenyang Pharmaceutical University, Shenyang 110016, People’s Republic of China

## Abstract

In the title compound, C_16_H_15_BrN_2_O_5_·2H_2_O, the dihedral angle between the two aromatic rings is 2.9 (2)° and an intra­molecular O—H⋯N hydrogen bond is observed. One of the water mol­ecule is disordered over two positions, with occupancies of 0.83 (3) and 0.17 (3). In the crystal structure, mol­ecules are linked into a three-dimensional network by inter­molecular O—H⋯O, O—H⋯(O,O), O—H⋯N and N—H⋯O hydrogen bonds. π–π inter­actions involving Br-substituted benzene rings, with a centroid–centroid distance of 3.552 (3) Å are also observed.

## Related literature

For related structures, see: Lu *et al.* (2008*a* ▶,*b* ▶,*c* ▶); Abdul Alhadi *et al.* (2009 ▶); Mohd Lair *et al.* (2009 ▶); Narayana *et al.* (2007 ▶). For bond-length data, see: Allen *et al.* (1987 ▶).
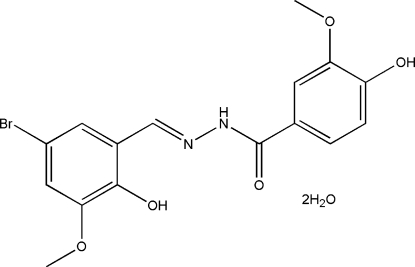


## Experimental

### 

#### Crystal data

C_16_H_15_BrN_2_O_5_·2H_2_O
*M* *_r_* = 431.24
Monoclinic, 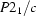

*a* = 9.262 (2) Å
*b* = 8.679 (2) Å
*c* = 24.289 (5) Å
β = 112.42 (3)°
*V* = 1804.9 (8) Å^3^
*Z* = 4
Mo *K*α radiation
μ = 2.32 mm^−1^
*T* = 298 K
0.23 × 0.20 × 0.20 mm

#### Data collection

Bruker APEXII CCD area-detector diffractometer
Absorption correction: multi-scan (*SADABS*; Sheldrick, 2004 ▶) *T*_min_ = 0.618, *T*_max_ = 0.654
14447 measured reflections
3897 independent reflections
1997 reflections with *I* > 2σ(*I*)
*R*_int_ = 0.074

#### Refinement

*R*[*F* ^2^ > 2σ(*F* ^2^)] = 0.051
*wR*(*F* ^2^) = 0.135
*S* = 1.02
3897 reflections
261 parameters
20 restraints
H atoms treated by a mixture of independent and constrained refinement
Δρ_max_ = 0.32 e Å^−3^
Δρ_min_ = −0.53 e Å^−3^

### 

Data collection: *APEX2* (Bruker, 2004 ▶); cell refinement: *SAINT* (Bruker, 2004 ▶); data reduction: *SAINT*; program(s) used to solve structure: *SHELXS97* (Sheldrick, 2008 ▶); program(s) used to refine structure: *SHELXL97* (Sheldrick, 2008 ▶); molecular graphics: *SHELXTL* (Sheldrick, 2008 ▶); software used to prepare material for publication: *SHELXTL*.

## Supplementary Material

Crystal structure: contains datablocks global, I. DOI: 10.1107/S1600536809033522/ci2888sup1.cif

Structure factors: contains datablocks I. DOI: 10.1107/S1600536809033522/ci2888Isup2.hkl

Additional supplementary materials:  crystallographic information; 3D view; checkCIF report

## Figures and Tables

**Table 1:** Hydrogen-bond geometry (Å, °)

*D*—H⋯*A*	*D*—H	H⋯*A*	*D*⋯*A*	*D*—H⋯*A*
O7—H7*B*⋯O1	0.85 (5)	2.34 (5)	2.875 (4)	122 (4)
O7—H7*B*⋯O2	0.85 (5)	2.22 (5)	3.027 (5)	159 (5)
O7—H7*A*⋯O6*A*	0.85 (5)	2.06 (2)	2.884 (10)	163 (6)
O6*A*—H6*B*⋯O7^i^	0.85 (1)	1.91 (4)	2.740 (8)	163 (5)
O6*A*—H6*A*⋯O3	0.85 (1)	1.92 (2)	2.715 (6)	154 (4)
N2—H2⋯O5^ii^	0.90	2.14	3.028 (4)	169
O5—H5⋯O6*B*^iii^	0.82	1.85	2.64 (3)	163
O5—H5⋯O6*A*^iii^	0.82	1.81	2.618 (5)	166
O1—H1⋯N1	0.82	1.83	2.550 (4)	145

Symmetry codes: (i) 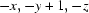; (ii) 
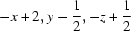
; (iii) 
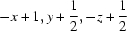
.

**Fig. 1.**
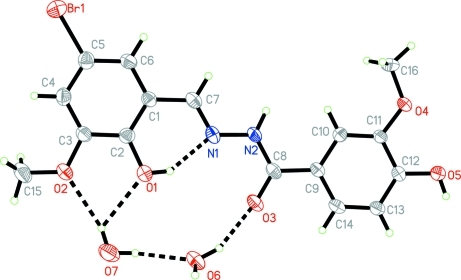
The molecular structure of the title compound, showing 30% probability displacement ellipsoids and the atom-numbering scheme. Hydrogen bonds are shown as dashed lines. Only the major component of a disordered water molecule is shown.

**Fig. 2.**
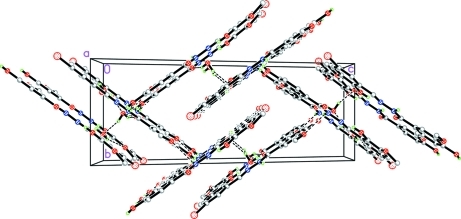
The crystal packing of the title compound, viewed along the a axis. H atoms not involved in hydrogen bonding (dashed lines) have been omitted for clarity.

## supplementary crystallographic information

### Comment

Schiff bases and their metal complexes have received much attention in recent years. As part of our investigation on the crystal structures of Schiff bases derived from the condensation of aldehydes with benzohydrazides (Lu *et al.*,  2008*a*,b,c), we report herein the crystal structure of the title new Schiff base compound.

The title compound (Fig. 1) consists of a Schiff base molecule and two water molecules of crystallization. The bond lengths have normal values (Allen *et al.*,  1987) and are comparable to those observed in related structures (Abdul Alhadi *et al.*, 2009; Mohd Lair *et al.*, 2009; Narayana *et al.*,  2007). The dihedral angle between the two aromatic rings is 2.9 (2)°, indicating that they are approximately coplanar. An intramolecular O—H···N hydrogen bond is observed (Fig. 1).

In the crystal structure, the molecules are linked into layers parallel to the ab direction by intermolecular N—H···O and O—H···O hydrogen bonds (Table 1 and Fig. 2).

### Experimental

The title compound was prepared by the Schiff base condensation of 5-bromo-2-hydroxy-3-methoxybenzaldehyde (0.1 mol) and 4-hydroxy-3-methoxybenzohydrazide (0.1 mmol) in 95% ethanol (50 ml). The excess ethanol was removed by distillation. The colourless solid obtained was filtered and washed with ethanol. Single crystals suitable for X-ray diffraction were obatined by slow evaporation of a 95% ethanol solution at room temperature.

### Refinement

One of the water oxygen (O6) is disordered over two positions (O6A and O6B) with occupancies of 0.83 (3) and 0.17 (3). The U^ij^ parameters of atoms O6B and O7 were restrained to an approximate isotropic behaviour. The H atoms of the water molecules were located in a difference map and refined with O-H and H···H distance restraints of 0.85 (1) and 1.37 (2) Å, respectively. The disordered water O atoms O6A and O6B share the same H atoms. All other H atoms were positioned geometrically (O-H = 0.82 Å and N-H = 0.90 Å and C-H = 0.93 or 0.96 Å) and refined using a riding model, with U_iso_(H) = 1.2U_eq_(C) and 1.5U_eq_(C_methyl_, O).

### Crystal data

**Table:**

C_16_H_15_BrN_2_O_5_·2H_2_O	*F*(000) = 880
*M**_r_* = 431.24	*D*_x_ = 1.587 Mg m^−^^3^
Monoclinic, *P*2_1_/*c*	Mo *K*α radiation, λ = 0.71073 Å
Hall symbol: -P 2ybc	Cell parameters from 1489 reflections
*a* = 9.262 (2) Å	θ = 2.4–24.5°
*b* = 8.679 (2) Å	µ = 2.32 mm^−^^1^
*c* = 24.289 (5) Å	*T* = 298 K
β = 112.42 (3)°	Block, colourless
*V* = 1804.9 (8) Å^3^	0.23 × 0.20 × 0.20 mm
*Z* = 4	

### Data collection

**Table:**

Bruker APEXII CCD area-detector diffractometer	3897 independent reflections
Radiation source: fine-focus sealed tube	1997 reflections with *I* > 2σ(*I*)
graphite	*R*_int_ = 0.074
ω scans	θ_max_ = 27.0°, θ_min_ = 1.8°
Absorption correction: multi-scan (*SADABS*; Sheldrick, 2004)	*h* = −11→11
*T*_min_ = 0.618, *T*_max_ = 0.654	*k* = −11→11
14447 measured reflections	*l* = −30→30

### Refinement

**Table:**

Refinement on *F*^2^	Primary atom site location: structure-invariant direct methods
Least-squares matrix: full	Secondary atom site location: difference Fourier map
*R*[*F*^2^ > 2σ(*F*^2^)] = 0.051	Hydrogen site location: inferred from neighbouring sites
*wR*(*F*^2^) = 0.135	H atoms treated by a mixture of independent and constrained refinement
*S* = 1.02	*w* = 1/[σ^2^(*F*_o_^2^) + 0.9035*P*] where *P* = (*F*_o_^2^ + 2*F*_c_^2^)/3
3897 reflections	(Δ/σ)_max_ = 0.001
261 parameters	Δρ_max_ = 0.32 e Å^−^^3^
20 restraints	Δρ_min_ = −0.53 e Å^−^^3^

### Special details

**Table:**

Geometry. All esds (except the esd in the dihedral angle between two l.s. planes) are estimated using the full covariance matrix. The cell esds are taken into account individually in the estimation of esds in distances, angles and torsion angles; correlations between esds in cell parameters are only used when they are defined by crystal symmetry. An approximate (isotropic) treatment of cell esds is used for estimating esds involving l.s. planes.
Refinement. Refinement of F^2^ against ALL reflections. The weighted R-factor wR and goodness of fit S are based on F^2^, conventional R-factors R are based on F, with F set to zero for negative F^2^. The threshold expression of F^2^ > 2sigma(F^2^) is used only for calculating R-factors(gt) etc. and is not relevant to the choice of reflections for refinement. R-factors based on F^2^ are statistically about twice as large as those based on F, and R- factors based on ALL data will be even larger.

### Fractional atomic coordinates and isotropic or equivalent isotropic displacement parameters (Å^2^)

**Table:**

	*x*	*y*	*z*	*U*_iso_*/*U*_eq_	Occ. (<1)
Br1	0.61788 (6)	−0.03732 (7)	−0.13511 (3)	0.0778 (3)	
O1	0.3146 (3)	0.3022 (4)	0.00769 (14)	0.0605 (9)	
H1	0.3776	0.3517	0.0351	0.091*	
O2	0.1468 (3)	0.1263 (4)	−0.07827 (14)	0.0692 (10)	
O3	0.4712 (3)	0.5527 (4)	0.14385 (13)	0.0601 (8)	
O4	1.1183 (3)	0.7762 (3)	0.28824 (13)	0.0570 (8)	
O5	0.9838 (3)	0.9110 (3)	0.35146 (13)	0.0504 (8)	
H5	0.9305	0.9520	0.3676	0.076*	
N1	0.5836 (4)	0.4024 (4)	0.07415 (15)	0.0448 (9)	
N2	0.6775 (4)	0.4898 (4)	0.12135 (14)	0.0446 (9)	
H2	0.7808	0.4791	0.1320	0.054*	
C1	0.5494 (5)	0.2381 (5)	−0.00796 (18)	0.0422 (10)	
C2	0.3905 (5)	0.2269 (5)	−0.02205 (19)	0.0450 (10)	
C3	0.3019 (5)	0.1318 (5)	−0.06863 (19)	0.0483 (11)	
C4	0.3704 (5)	0.0548 (5)	−0.10141 (19)	0.0516 (11)	
H4	0.3102	−0.0075	−0.1330	0.062*	
C5	0.5288 (5)	0.0694 (5)	−0.08767 (19)	0.0515 (11)	
C6	0.6186 (5)	0.1586 (5)	−0.04129 (18)	0.0477 (11)	
H6	0.7255	0.1665	−0.0319	0.057*	
C7	0.6451 (5)	0.3319 (5)	0.04189 (19)	0.0476 (11)	
H7	0.7516	0.3412	0.0506	0.057*	
C8	0.6112 (5)	0.5611 (5)	0.15561 (18)	0.0434 (10)	
C9	0.7141 (4)	0.6511 (4)	0.20645 (17)	0.0392 (10)	
C10	0.8737 (5)	0.6677 (4)	0.22134 (18)	0.0421 (10)	
H10	0.9213	0.6195	0.1985	0.050*	
C11	0.9612 (4)	0.7545 (4)	0.26950 (18)	0.0395 (10)	
C12	0.8909 (5)	0.8267 (4)	0.30448 (17)	0.0389 (10)	
C13	0.7334 (5)	0.8129 (5)	0.28971 (18)	0.0468 (11)	
H13	0.6856	0.8622	0.3123	0.056*	
C14	0.6469 (5)	0.7260 (5)	0.24137 (19)	0.0476 (11)	
H14	0.5400	0.7168	0.2316	0.057*	
C15	0.0554 (6)	0.0149 (6)	−0.1176 (2)	0.0779 (16)	
H15A	0.0466	0.0389	−0.1573	0.117*	
H15B	−0.0468	0.0134	−0.1161	0.117*	
H15C	0.1033	−0.0844	−0.1063	0.117*	
C16	1.1985 (5)	0.7209 (6)	0.2527 (2)	0.0680 (14)	
H16A	1.1888	0.6109	0.2493	0.102*	
H16B	1.3069	0.7484	0.2709	0.102*	
H16C	1.1539	0.7662	0.2137	0.102*	
O6A	0.1601 (5)	0.5145 (11)	0.0818 (2)	0.061 (3)	0.83 (3)
H6A	0.248 (2)	0.553 (4)	0.1037 (19)	0.091*	
H6B	0.108 (4)	0.591 (4)	0.0621 (18)	0.091*	
O6B	0.161 (3)	0.596 (5)	0.0998 (16)	0.071 (10)	0.17 (3)
O7	−0.0002 (3)	0.2697 (5)	0.00144 (17)	0.0817 (11)	
H7A	0.055 (5)	0.327 (6)	0.0300 (19)	0.123*	
H7B	0.059 (5)	0.221 (6)	−0.012 (2)	0.123*	

### Atomic displacement parameters (Å^2^)

**Table:**

	*U*^11^	*U*^22^	*U*^33^	*U*^12^	*U*^13^	*U*^23^
Br1	0.0656 (4)	0.0946 (5)	0.0795 (4)	0.0048 (3)	0.0346 (3)	−0.0224 (3)
O1	0.0426 (18)	0.072 (2)	0.063 (2)	−0.0067 (16)	0.0166 (16)	−0.0216 (17)
O2	0.0376 (18)	0.089 (2)	0.078 (2)	−0.0146 (18)	0.0190 (17)	−0.030 (2)
O3	0.0332 (17)	0.078 (2)	0.064 (2)	−0.0127 (16)	0.0128 (15)	−0.0105 (17)
O4	0.0299 (16)	0.070 (2)	0.069 (2)	−0.0099 (15)	0.0168 (15)	−0.0251 (16)
O5	0.0427 (17)	0.058 (2)	0.0527 (19)	−0.0048 (15)	0.0208 (15)	−0.0106 (15)
N1	0.038 (2)	0.045 (2)	0.042 (2)	−0.0051 (17)	0.0047 (17)	0.0042 (17)
N2	0.0273 (17)	0.053 (2)	0.045 (2)	−0.0014 (16)	0.0042 (16)	−0.0033 (17)
C1	0.037 (2)	0.042 (3)	0.044 (3)	0.002 (2)	0.011 (2)	0.003 (2)
C2	0.045 (3)	0.042 (3)	0.049 (3)	0.002 (2)	0.019 (2)	0.000 (2)
C3	0.037 (2)	0.049 (3)	0.056 (3)	−0.002 (2)	0.015 (2)	−0.006 (2)
C4	0.051 (3)	0.048 (3)	0.050 (3)	−0.003 (2)	0.013 (2)	−0.008 (2)
C5	0.051 (3)	0.050 (3)	0.049 (3)	0.002 (2)	0.014 (2)	0.000 (2)
C6	0.037 (2)	0.058 (3)	0.047 (3)	0.001 (2)	0.015 (2)	0.005 (2)
C7	0.036 (2)	0.052 (3)	0.048 (3)	−0.001 (2)	0.009 (2)	0.007 (2)
C8	0.034 (2)	0.049 (3)	0.041 (2)	−0.003 (2)	0.007 (2)	0.010 (2)
C9	0.032 (2)	0.041 (2)	0.041 (2)	−0.0017 (18)	0.0101 (19)	0.0039 (19)
C10	0.041 (2)	0.039 (3)	0.046 (3)	0.0000 (19)	0.017 (2)	−0.003 (2)
C11	0.032 (2)	0.038 (2)	0.049 (3)	−0.0019 (19)	0.016 (2)	−0.001 (2)
C12	0.037 (2)	0.039 (2)	0.038 (2)	−0.0026 (19)	0.011 (2)	0.0047 (19)
C13	0.038 (2)	0.056 (3)	0.049 (3)	−0.004 (2)	0.019 (2)	−0.004 (2)
C14	0.032 (2)	0.055 (3)	0.057 (3)	−0.002 (2)	0.018 (2)	0.005 (2)
C15	0.049 (3)	0.082 (4)	0.092 (4)	−0.018 (3)	0.016 (3)	−0.016 (3)
C16	0.040 (3)	0.087 (4)	0.084 (4)	0.003 (3)	0.031 (3)	−0.023 (3)
O6A	0.037 (3)	0.081 (5)	0.056 (3)	−0.009 (2)	0.010 (2)	0.009 (3)
O6B	0.062 (12)	0.077 (14)	0.074 (13)	0.001 (8)	0.025 (9)	0.026 (8)
O7	0.048 (2)	0.112 (3)	0.083 (3)	0.000 (2)	0.0222 (18)	−0.002 (2)

### Geometric parameters (Å, °)

**Table:**

Br1—C5	1.895 (4)	C8—C9	1.465 (5)
O1—C2	1.353 (5)	C9—C10	1.389 (5)
O1—H1	0.82	C9—C14	1.390 (5)
O2—C3	1.365 (5)	C10—C11	1.367 (5)
O2—C15	1.396 (5)	C10—H10	0.93
O3—C8	1.218 (5)	C11—C12	1.400 (5)
O4—C11	1.361 (4)	C12—C13	1.368 (5)
O4—C16	1.421 (5)	C13—C14	1.368 (6)
O5—C12	1.353 (4)	C13—H13	0.93
O5—H5	0.82	C14—H14	0.93
N1—C7	1.286 (5)	C15—H15A	0.96
N1—N2	1.373 (4)	C15—H15B	0.96
N2—C8	1.357 (5)	C15—H15C	0.96
N2—H2	0.90	C16—H16A	0.96
C1—C2	1.382 (5)	C16—H16B	0.96
C1—C6	1.392 (5)	C16—H16C	0.96
C1—C7	1.448 (6)	O6A—O6B	0.83 (4)
C2—C3	1.388 (6)	O6A—H6A	0.851 (10)
C3—C4	1.367 (6)	O6A—H6B	0.853 (10)
C4—C5	1.381 (6)	O6B—H6A	0.856 (10)
C4—H4	0.93	O6B—H6B	0.858 (10)
C5—C6	1.358 (6)	O7—H7A	0.85 (5)
C6—H6	0.93	O7—H7B	0.85 (5)
C7—H7	0.93		
			
C2—O1—H1	109.5	C11—C10—C9	120.3 (4)
C3—O2—C15	117.8 (4)	C11—C10—H10	119.8
C11—O4—C16	119.3 (3)	C9—C10—H10	119.8
C12—O5—H5	109.5	O4—C11—C10	124.8 (4)
C7—N1—N2	119.0 (3)	O4—C11—C12	114.8 (3)
C8—N2—N1	118.3 (3)	C10—C11—C12	120.3 (4)
C8—N2—H2	123.6	O5—C12—C13	122.7 (4)
N1—N2—H2	116.8	O5—C12—C11	117.4 (3)
C2—C1—C6	120.3 (4)	C13—C12—C11	119.9 (4)
C2—C1—C7	120.1 (4)	C14—C13—C12	119.3 (4)
C6—C1—C7	119.6 (4)	C14—C13—H13	120.3
O1—C2—C1	123.7 (4)	C12—C13—H13	120.3
O1—C2—C3	117.2 (4)	C13—C14—C9	122.0 (4)
C1—C2—C3	119.1 (4)	C13—C14—H14	119.0
O2—C3—C4	125.1 (4)	C9—C14—H14	119.0
O2—C3—C2	114.6 (4)	O2—C15—H15A	109.5
C4—C3—C2	120.3 (4)	O2—C15—H15B	109.5
C3—C4—C5	120.0 (4)	H15A—C15—H15B	109.5
C3—C4—H4	120.0	O2—C15—H15C	109.5
C5—C4—H4	120.0	H15A—C15—H15C	109.5
C6—C5—C4	120.7 (4)	H15B—C15—H15C	109.5
C6—C5—Br1	120.8 (3)	O4—C16—H16A	109.5
C4—C5—Br1	118.5 (3)	O4—C16—H16B	109.5
C5—C6—C1	119.5 (4)	H16A—C16—H16B	109.5
C5—C6—H6	120.2	O4—C16—H16C	109.5
C1—C6—H6	120.2	H16A—C16—H16C	109.5
N1—C7—C1	120.3 (4)	H16B—C16—H16C	109.5
N1—C7—H7	119.8	O6B—O6A—H6A	61.2 (18)
C1—C7—H7	119.8	O6B—O6A—H6B	61.3 (17)
O3—C8—N2	121.2 (4)	H6A—O6A—H6B	104 (2)
O3—C8—C9	121.5 (4)	O6A—O6B—H6A	60.6 (18)
N2—C8—C9	117.3 (4)	O6A—O6B—H6B	60.6 (18)
C10—C9—C14	118.2 (4)	H6A—O6B—H6B	103 (2)
C10—C9—C8	124.1 (4)	H7A—O7—H7B	109 (3)
C14—C9—C8	117.7 (4)		
			
C7—N1—N2—C8	−178.8 (4)	N1—N2—C8—O3	−2.5 (6)
C6—C1—C2—O1	−179.1 (4)	N1—N2—C8—C9	178.8 (3)
C7—C1—C2—O1	1.3 (6)	O3—C8—C9—C10	−178.6 (4)
C6—C1—C2—C3	2.0 (6)	N2—C8—C9—C10	0.2 (6)
C7—C1—C2—C3	−177.6 (4)	O3—C8—C9—C14	0.5 (6)
C15—O2—C3—C4	11.5 (7)	N2—C8—C9—C14	179.2 (3)
C15—O2—C3—C2	−169.8 (4)	C14—C9—C10—C11	0.6 (6)
O1—C2—C3—O2	−0.2 (6)	C8—C9—C10—C11	179.7 (4)
C1—C2—C3—O2	178.8 (4)	C16—O4—C11—C10	7.5 (6)
O1—C2—C3—C4	178.6 (4)	C16—O4—C11—C12	−173.8 (4)
C1—C2—C3—C4	−2.4 (6)	C9—C10—C11—O4	179.1 (4)
O2—C3—C4—C5	179.8 (4)	C9—C10—C11—C12	0.4 (6)
C2—C3—C4—C5	1.1 (7)	O4—C11—C12—O5	1.3 (5)
C3—C4—C5—C6	0.7 (7)	C10—C11—C12—O5	−179.9 (3)
C3—C4—C5—Br1	−179.1 (3)	O4—C11—C12—C13	179.8 (3)
C4—C5—C6—C1	−1.1 (6)	C10—C11—C12—C13	−1.4 (6)
Br1—C5—C6—C1	178.7 (3)	O5—C12—C13—C14	179.7 (4)
C2—C1—C6—C5	−0.2 (6)	C11—C12—C13—C14	1.2 (6)
C7—C1—C6—C5	179.3 (4)	C12—C13—C14—C9	−0.2 (6)
N2—N1—C7—C1	179.6 (3)	C10—C9—C14—C13	−0.8 (6)
C2—C1—C7—N1	1.6 (6)	C8—C9—C14—C13	−179.9 (4)
C6—C1—C7—N1	−178.0 (4)		

### Hydrogen-bond geometry (Å, °)

**Table:**

*D*—H···*A*	*D*—H	H···*A*	*D*···*A*	*D*—H···*A*
O7—H7B···O1	0.85 (5)	2.34 (5)	2.875 (4)	122 (4)
O7—H7B···O2	0.85 (5)	2.22 (5)	3.027 (5)	159 (5)
O7—H7A···O6A	0.85 (5)	2.06 (2)	2.884 (10)	163 (6)
O6A—H6B···O7^i^	0.85 (1)	1.91 (4)	2.740 (8)	163 (5)
O6A—H6A···O3	0.85 (1)	1.92 (2)	2.715 (6)	154 (4)
N2—H2···O5^ii^	0.90	2.14	3.028 (4)	169
O5—H5···O6B^iii^	0.82	1.85	2.64 (3)	163
O5—H5···O6A^iii^	0.82	1.81	2.618 (5)	166
O1—H1···N1	0.82	1.83	2.550 (4)	145

Symmetry codes: (i) −*x*, −*y*+1, −*z*; (ii) −*x*+2, *y*−1/2, −*z*+1/2; (iii) −*x*+1, *y*+1/2, −*z*+1/2.
